# Multilayer Coating with Red Ginseng Dietary Fiber Improves Intestinal Adhesion and Proliferation of Probiotics in Human Intestinal Epithelial Models

**DOI:** 10.4014/jmb.2305.05013

**Published:** 2023-07-14

**Authors:** Ye Seul Son, Mijin Kwon, Naeun Son, Sang-Kyu Kim, Mi-Young Son

**Affiliations:** 1 Korea Research Institute of Bioscience and Biotechnology (KRIBB), Daejeon 34141, Republic of Korea; 2 KRIBB School of Bioscience, Korea University of Science and Technology, Daejeon 34113, Republic of Korea; 3 Laboratory of Efficacy Research, Korea ginseng Corporation, Daejeon 34128, Republic of Korea; 4 Department of Biological Science, Sungkyunkwan University, Suwon, 16419, Republic of Korea

**Keywords:** Multilayer coating, red ginseng dietary fiber, adhesion, acid tolerance, in vitro digestive system, human intestinal epithelial cells

## Abstract

To exert their beneficial effects, it is essential for the commensal bacteria of probiotic supplements to be sufficiently protected as they pass through the low pH environment of the stomach, and effectively colonize the intestinal epithelium downstream. Here, we investigated the effect of a multilayer coating containing red ginseng dietary fiber, on the acid tolerance, and the adhesion and proliferation capacities of three *Lactobacillus* strains (*Limosilactobacillus reuteri* KGC1901, *Lacticaseibacillus casei* KGC1201, *Limosilactobacillus fermentum* KGC1601) isolated from *Panax ginseng*, using HT-29 cells, mucin-coated plates, and human pluripotent stem cell-derived intestinal epithelial cells as in vitro models of human gut physiology. We observed that the multilayer-coated strains displayed improved survival rates after passage through gastric juice, as well as high adhesion and proliferation capacities within the various gut epithelial systems tested, compared to their uncoated counterparts. Our findings demonstrated that the multilayer coat effectively protected commensal microbiota and led to improved adhesion and colonization of intestinal epithelial cells, and consequently to higher probiotic efficacy.

## Introduction

Probiotics are defined as “living microorganisms that help host health when consumed in appropriate amounts,” and have been studied for their various positive effects on the host [1]. Probiotics can reportedly contribute to the regulation of the immune response, the maintenance of intestinal barrier integrity, as well as the prevention and inhibition of harmful bacteria colonization, and the reduction of fat accumulation [2] Various probiotic strains have been commercialized as nutritional supplements and foods [3] and are now being studied as potential therapeutics for managing gastrointestinal disorders [4].

Probiotic supplements are available in many different forms, such as capsules, powders, and beverages, and most of these preparation methods require freeze-drying (lyophilizing) of the probiotics [5, 6]. During the freeze-drying process, probiotics are subjected to dehydration shock, that can impair cell-surface function by destabilizing membrane components, leading to loss of the lipid bilayer integrity [7, 8]. The lyophilization-mediated damage of the cell surface can negatively affect the adhesion properties of cells. As such, a more efficient cytoprotective coating of cells can in turn result in high adhesion and survival capacity of the ingested probiotics, allowing these to successfully colonize the intestinal epithelium [9, 10].

Lactic acid bacteria (LAB), which are well-known and generally regarded as safe probiotics, must first survive after passing through the harsh gastric environment, and then adhere to the intestinal mucosa, establish viable colonies, and interact with intestinal cells or microorganisms via the production of metabolites [11, 12]. Following oral administration, although LAB pass through the gastric juice and reach the intestinal tract, their adhesion ability is reduced and as a result, most are excreted in feces [13]. Therefore, an important factor during intake of probiotics is their prolonged survival under the extreme acidic conditions of the stomach and their successful surface adhesion and colonization of the intestinal epithelium.

Recently, we reported that multilayer coating methods that include a key coating step of encapsulating LAB by red ginseng dietary fiber (RGDF), can enhance the acid tolerance, viability, and adhesion of the encapsulated commensal bacteria [2]. However, it is also necessary to utilize an appropriate in vitro intestinal epithelium model that more closely mimics the environment of the human intestine when examining the temporal adhesion and long-term gut colonization by LAB. To this end, we have developed functional human intestinal epithelial cells (hIECs) derived from human pluripotent stem cells (hPSCs), which display normal human gut epithelium characteristics [14], such as apical-basolateral polarity and drug-metabolizing activity, and comprising of various intestinal cell types, including enterocytes, Paneth, mucus-producing goblet, and hormone-secreting enteroendocrine cells, as well as dense microvilli. The goblet cells contained in the hIECs express genes related to mucus production, which facilitate colonization by LAB [14].

In this study, we aimed to analyze the effects of an RGDF-based multilayer coating on LAB adhesion and acid tolerance in a consecutive in vitro digestive system, by modelling gastric juice and the intestinal epithelium microenvironment using mucin-coated well plates, a conventional HT-29 colon cell line, and hPSC-derived normal hIECs.

## Materials and Methods

### Bacterial Strain, Culture Condition, and Coating

The bacterial strains used in this study were *Limosilactobacillus reuteri* KGC1901 (deposit number KCTC 14652BP) [15], *Lacticaseibacillus casei* KGC1201 (deposit number KCTC 14651BP) [16], and *Limosilactobacillus fermentum* KGC1601 (deposit number KCTC1473BP) [17]. All the strains isolated from *Panax ginseng* were obtained from the Korean Ginseng Corporation. The probiotics were cultured at 37°C for 18 h in sterilized de Man, Rogosa, and Sharpe (MRS) agar (BD Biosciences, USA). To apply the multilayer coat, strains were centrifuged at 8,000 ×*g* for 3 min and coated with tara gum, basic amino acids (including L-histidine, L-arginine, and L-lysine), rice protein powder, and RGDF, which were lyophilized, as per the method described by Jeon *et al*. [2]. The uncoated strains (UCS) were lyophilized after centrifugation. All strains were used at approximately equal amounts, by measuring the number of bacteria per 1 g via the plate count method [18].

### Mammalian Cell Culture

The adhesion ability of the bacteria was measured in HT-29 cells and hPSC-derived hIECs. The cell lines were cultured in Roswell Park Memorial Institute (RPMI) 1640 medium (Cytiva, USA) with 10% fetal bovine serum (FBS; HyClone, USA) and 1% penicillin/streptomycin (PS; Gibco, USA) at 37°C in a humidified incubator with 5% CO_2_.

The hPSC line (H9) was purchased from the WiCell Research Institute (Madison, USA). The hPSCs were cultured on Mitomycin C-treated mouse embryonic fibroblasts (MMC-MEF) using hPSC medium [Dulbecco’s modified Eagle’s medium (DMEM)/F12 (Gibco), 10% serum replacement (SR, Gibco), 1% PS (Gibco), 1%GlutaMAX (Gibco), 0.1% β-mercaptoethanol (Gibco), and 8 ng/ml basic fibroblast growth factor (bFGF; R&D Systems, USA)]. The hPSCs were passaged every week to newly prepared MMC-MEF [19]. All experiments were approved by the Public Institutional Review Board designated by the Ministry of Health and Welfare (P01-201409-ES-01).

### Differentiation of hPSCs to Functional hIECs

The hPSCs were differentiated into hIECs as previously described [14]. To induce formation of the definitive endoderm, the hPSCs were treated with 100 ng/ml activin A (R&D Systems, USA) for three days in RPMI-1640 medium (Gibco) containing 0, 0.2, or 2% FBS. The cells were then further differentiated into hindgut tissue by treatment with 250 ng/ml fibroblast growth factor 4 (Peprotech, USA) and 1.2 μM CHIR99021 (Tocris Bioscience, USA) in DMEM/F12 (Gibco) supplemented with 2% FBS. The cells were reseeded onto a 1% Matrigel-coated tissue culture plate for differentiation into hIEC progenitors and cultured in hIEC-differentiation medium 1 (hIEC medium 1) containing DMEM/F12, 100 ng/ml epithelial growth factor (EGF) (R&D Systems), 5 μg/ml insulin (Thermo Fisher Scientific Inc. USA), 100 ng/ml R-spondin 1 (Peprotech), 2% FBS, 1 × N_2_ supplement (Gibco), 1 × B27 supplement (Gibco), 2 mM l-glutamine (Gibco), 1% NEAA, and 15 mM HEPES buffer (Gibco). The hIEC medium 1 was replaced every two days, and the hIEC progenitors were passaged every seven days. To differentiate the hIEC progenitors into functional hIECs, 1.34 × 10^5^ cells/cm^2^ hIEC progenitors were reseeded onto 1% Matrigel-coated Transwell (Corning) inserts with hIEC medium 1 containing 10 μM Y-27632 (Tocris) and incubated for the first two days. At day 3, the medium was replaced with hIEC differentiation medium 2 (hIEC medium 2) containing DMEM/F12, 100 ng/ml EGF, 2 μM Wnt-C59 (Selleckchem, USA), 1 mM valproic acid (Stemgent, USA) acid, 2% FBS, 1 × N_2_ supplement, 1 × B27 supplement, 2 mM L-glutamine, 1% NEAA, and 15 mM HEPES buffer, for differentiation into functional hIECs. The hIECs were cultured for 10–14 days prior to analysis.

### Adhesion Assay

To prepare a 0.4% mucin solution, mucin from porcine stomach type II (Sigma-Aldrich, Germany) was diluted in a carbohydrate coating buffer. The solution was dispensed into a 12- well non-coated plate (SPL, Korea), and stored at 4°C for 24 h to allow formation of a mucin coating. To test the LAB strains’ retention and proliferation capacity as a result of the increased adhesion ability, cells were divided into 2 h- and 24 h-adhesion groups. The UCS and the coated strains (CS) were inoculated at 10^7^ CFU/ml in RPMI-1640, into mucin-coated wells and maintained for 2 h in an incubator with a humidified atmosphere of 5% CO_2_ at 37°C. To remove non-adherent cells, the wells were washed with PBS. To measure the number of the adhesion group, the bacteria were treated with 0.5% trypsin-EDTA for 3 min to separate from the mucin-coated well plate and subsequently counted after incubation at 37° C for 48 h in MRS agar. The 24 h-group was cultured in fresh medium for 24 h, and the number of viable cells was subsequently measured.

The HT-29 cells were seeded for 24 h at a density of 2.0 × 10^5^ cells in a 12-well plate using medium without PS. Functional hIECs prepared on the Transwell were maintained using hIEC medium 2 without PS. The probiotics were inoculated at 10^7^ CFU/ml in each cell culture medium and maintained for 2 h. Strains were washed and treated with trypsin-EDTA to measure cell number, or further cultured in fresh medium to examine the proliferation of the adherent strains after 24 h. The number of viable probiotics was determined as described above.

### Immunofluorescence Staining

The HT-29 cells were inoculated in 2-well cell culture slides (SPL) at a density of 5.0 × 10^5^ cells/well and incubated for 24 h. In regard to hIECs, the progenitor cells were re-seeded onto a 1% Matrigel coated Transwell at a concentration of 1.34 × 10^5^ cells/cm^2^, and maintained with hIEC medium 2 for differentiation into functional hIECs. The UCS and CS were inoculated into HT-29 cells or functional hIECs, at a count of 1.0 × 10^6^ CFU and 1.0 × 10^7^ CFU, respectively, and further incubated for 2 h. Cells were washed with PBS and fixed with 4%paraformaldehyde. After permeabilization with 0.1% Triton X-100 (Sigma-Aldrich), the cells were blocked in 4%bovine serum albumin (Sigma-Aldrich) for 1 h, prior to incubating with a primary mouse anti-peptidoglycan antibody, and subsequent staining with a fluorescent secondary goat anti-mouse IgG H&L Alexa Fluor 488 antibody (Invitrogen, USA). The HT-29 cells were mounted on coverslips using mounting medium with DAPI (Abcam, UK). The hIECs were stained with DAPI (Thermo Fisher Scientific Inc.) and mounted using fluorescence mounting medium (Dako, Japan). Cells were imaged using an LSM 800 confocal laser scanning microscope (Carl Zeiss, Germany). The antibodies used are listed in Supplementary Materials.

### Acid Tolerance Test and Consecutive Bacterial Adhesion Assay

To measure the cytoprotective capacity of the multilayer coat against gastric acid and its subsequent effect on LAB adhesion ability on intestinal epithelium cells, LAB strains were passed through a gastric juice-simulating acid solution. Acid tolerance test was performed via a modified version of the method described by Lee [16]. The bacteria were treated with PBS (pH 2.5) for 3 h at a density of 10^7^ CFU and washed with PBS. After measuring the number of viable strains, they were inoculated into plates with mucin, HT-29 cells, or hIECs. The adhesive capacities of the UCS and CS were evaluated as previously described.

### Statistical Analysis

All values were obtained from minimum three independent experiments and are shown as mean ± standard deviation. Group differences were statistically analyzed using an unpaired t-test, and the level of significance was considered as: * *p* < 0.05, ** *p* < 0.01, and *** *p* < 0.001.

## Results

### Increased Adhesion Ability of LAB by RGDF-Containing Multilayer Coating in HT-29 Cells and Mucin-Coated Plates

**Fig. 1:**
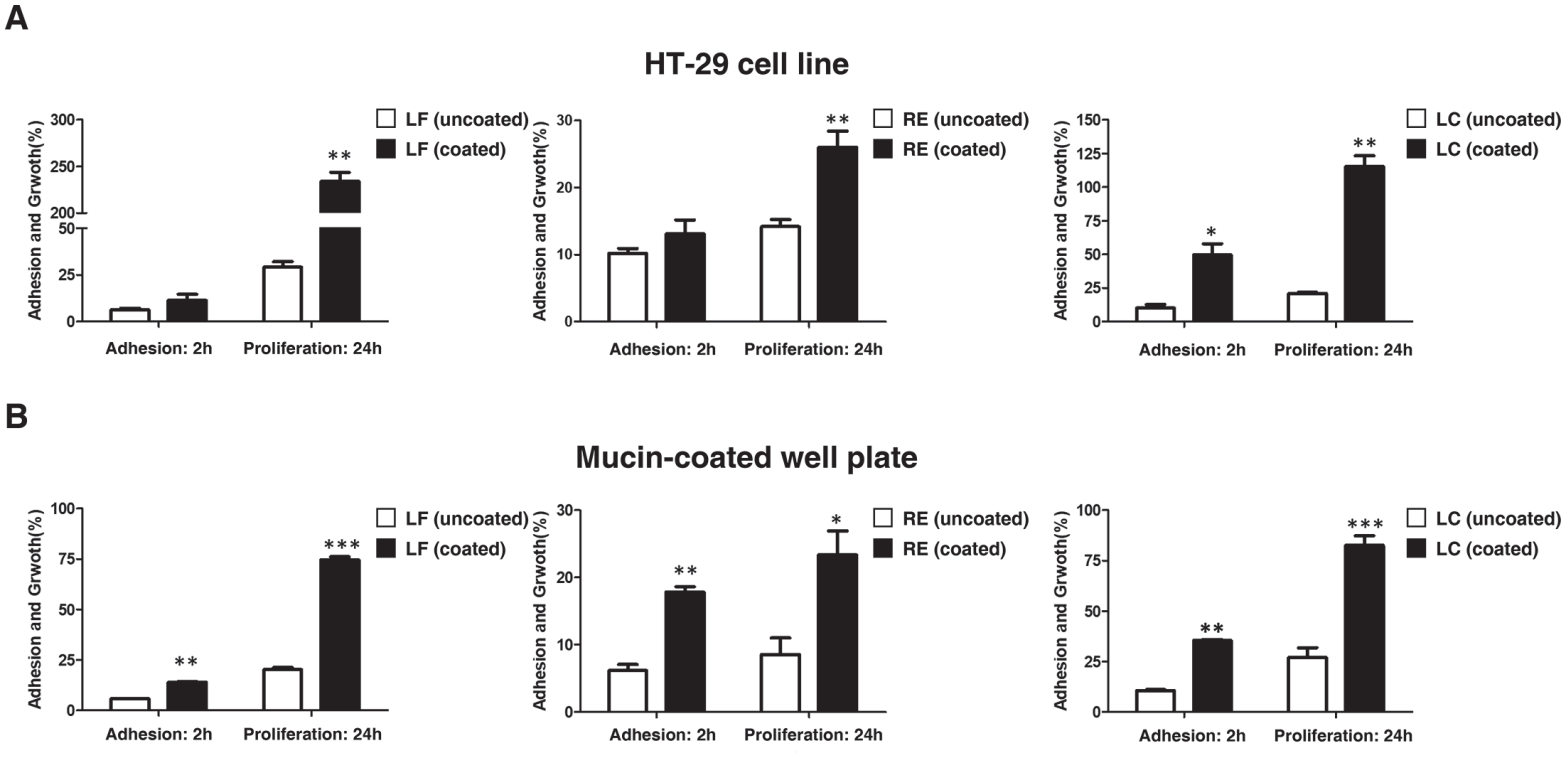
Effect of multilayer coating on adhesion ability and proliferation of LAB strains in HT-29 cell lines and mucin-coated well plates. Comparison of adhesion ability in relation to the initial number of LF, RE, and LC at adhesion (2 h) and proliferation (24 h) in (**A**) HT-29 cell lines, and (**B**) mucin-coated well plates. Groups were compared using an unpaired *t*-test (*n* = 4; *p<0.05, **p < 0.01 and ***p < 0.001).

To examine if the multilayer coating using RGDF increases the adhesive ability of LAB, we inoculated uncoated and coated *L. fermentum* KGC1601 (LF), *L. reuteri* KGC1901 (RE), *L. casei* KGC1201 (LC) into HT-29 cells or mucin-coated well-plates and incubated samples for 2 h. We observed that immediately after the 2 h mark, the adhesive ability of CS was higher than that of UCS in HT-29 cells (Fig. 1A). Adhesion of coated-LC was significantly increased by the coating method, compared to uncoated-LC (4.92-fold difference; Fig. 1A). After 24 h, the number of bacteria in all strains had increased more in relation to the initial number of adherent bacteria. The adhesion abilities of the LF, RE, and LC strains in the UCS group were 29.2%, 14.2%, and 20.9%, respectively, while the corresponding ones in the CS group were 234.1% (LF), 26.0% (RE), and 115.5% (LC) (Fig. 1A). In the mucin-coated well plate, we observed proliferation in all strains after 24 h compared to the initial adhesion period (Fig. 1B). Interestingly, the adhesive ability of the CS group was found to be consistently elevated by a significant margin relative to the UCS group. In particular, the proliferation ability of LF and LC in the CS group after 24 h was 74.7% and 82.6%, respectively, which was higher than the corresponding values of 50% (LF) and 25% (LC) of the UCS counterparts. These results suggest that the RGDF-based multilayer coating improved the adhesion capacity and proliferation of LAB strains in both HT-29 cells and the mucin-covered plate.

**Fig. 2:**
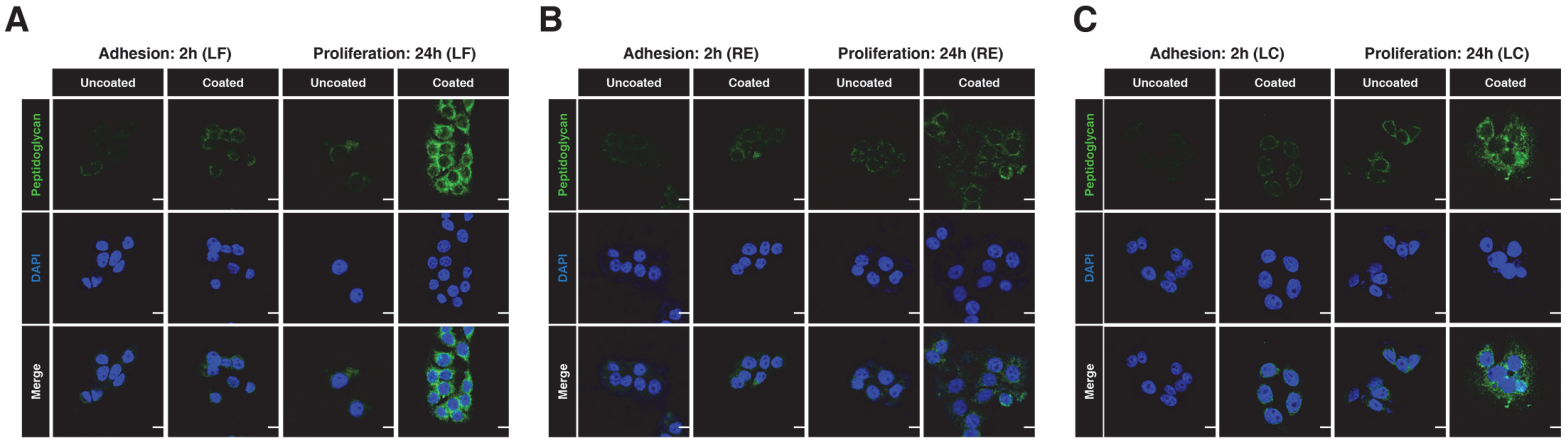
Florescence image analysis of the multilayer coating effect on LAB strains adhesion and proliferation in HT-29 cell lines. Representative fluorescence images of (**A**) LF, (**B**) RE, and (**C**) LC at adhesion (2 h) and proliferation (24 h) in HT-29 cell lines. Green fluorescence indicates bacterial peptidoglycan. Scale bar: 20 μm.

To visualize the adhesion and proliferation of the LF, RE, and LC strains, we performed immunofluorescence imaging of the HT-29 cell line. After 2 h of attachment and subsequent PBS wash steps, the fluorescence images showed that coated-LF adhered slightly more than uncoated LF (Fig. 2A, left panels). After a further 24 h incubation, coated-LF seemed to adhere and proliferate noticeably more than uncoated-LF (Fig. 2A, right panels). Similarly, Fig. 2B depicts the adhesion and proliferation differences between coated- and uncoated-RE. Based on our image comparison, the initial adhesion ability after 2 h of attachment was somewhat similar between coated-and uncoated-RE (Fig. 2B, left panels), however this was distinctly higher for coated-RE after 24 h of incubation (Fig. 2B, right panels). In a similar manner, coated-LC also displayed evidence of improved adhesion and proliferation capability compared to uncoated-LC (Fig. 2C, right panels), although the initial adhesion ability was comparable between the two conditions (Fig. 2C, left panels). Together, the fluorescence imaging results indicated that the RGDF-based multilayer coating increased the adhesion and proliferation capacity of LAB strains, which further supported our biochemical observations.

### Increased Acid Resistance and Consecutive Bacterial Adhesion Capacity

**Fig. 3:**
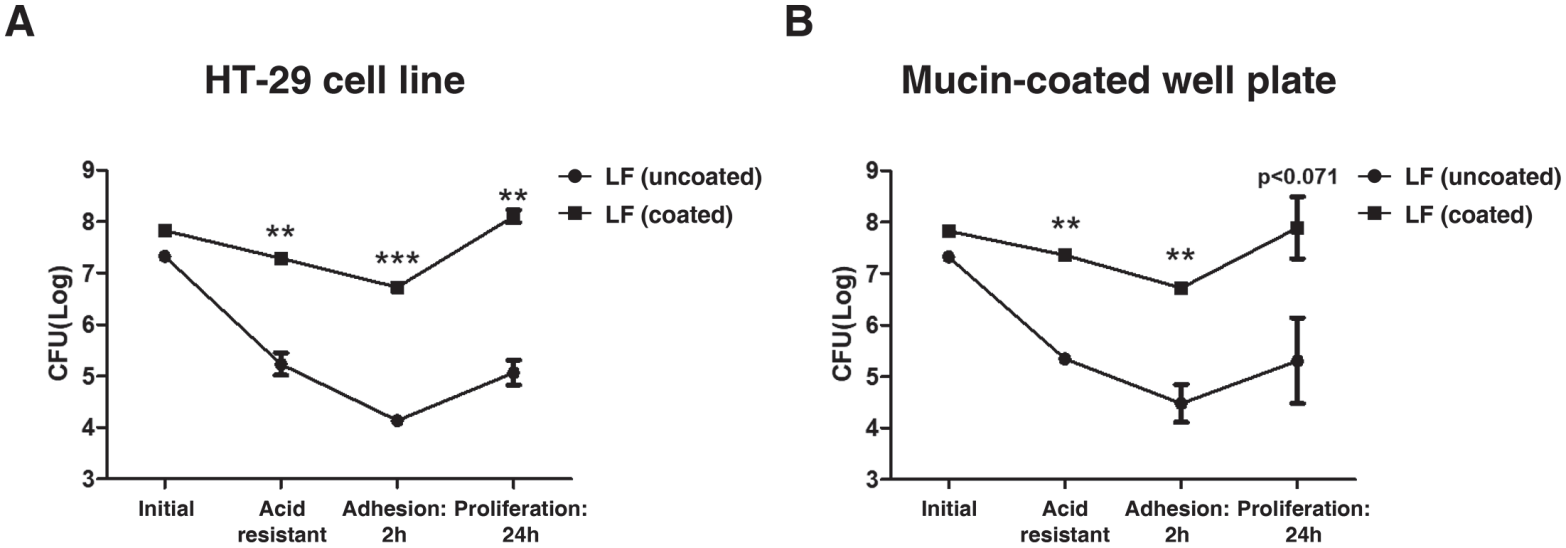
Acid resistance and consecutive bacterial adhesion. Analysis of adhesion (2 h) ability and proliferation (24 h) of LF after passing through gastric juice in (**A**) HT-29 cell lines, and (**B**) mucin-coated well plates. Groups were compared using an unpaired *t*-test (*n* = 4; *p<0.05, **p < 0.01 and ***p < 0.001).

To examine whether the adhesion capacity of LF can be increased by the RGDF-based multilayer coating after passing through the stomach’s harsh acidic conditions, we treated the strains with gastric fluid for 3 h and then measured the number of adhering bacteria on the gut epithelial surface at the 2 h- and 24 h-mark. Each strain exposed to acidic conditions was inoculated into HT-29 cells and cultured for 2 h to test the adhesion capacity. The number of viable uncoated- and coated-LF in the presence of gastric juice decreased from 6.70 × 10^5^ to 1.99 × 10^7^, and from 2.12 × 10^5^ to 1.82 × 10^5^, respectively, (Fig. 3A). Subsequently, the uncoated- and coated-LF attached to HT-29 cells at a bacterial count of 1.40 × 10^4^ and 5.43 × 10^5^, respectively. After the adhesive strains were further cultured for 24 h, the viable cell number of adhered coated-LF was found to be higher than uncoated-LF (1.30 × 10^7^ vs. 1.26 × 10^5^) and corresponded to a 103.17% increase (Fig. 3A). Coated-LF also demonstrated higher acid resistance compared to uncoated-LF in mucin-coated well plates, as well as a 100-fold increase in adhesion ability (Fig. 2B). In addition, the number of viable bacteria after 2 h of incubation was significantly higher in the coated-LF group compared to uncoated-LF, which was also mirrored in the increased proliferation of coated-LF observed after 24 h of culture (*p* < 0.071; Fig. 3B). Overall, these findings suggest that the RGDF-based multilayer coating protected LF during passage through the simulated acidic environment of the stomach, and allowed for improved adherence and proliferation in the intestine, as indicated by our conventional intestine model systems.

### Increased Acid Resistance and Consecutive Bacterial Adhesion in a Normal Human Intestinal Epithelium Model

**Fig. 4:**
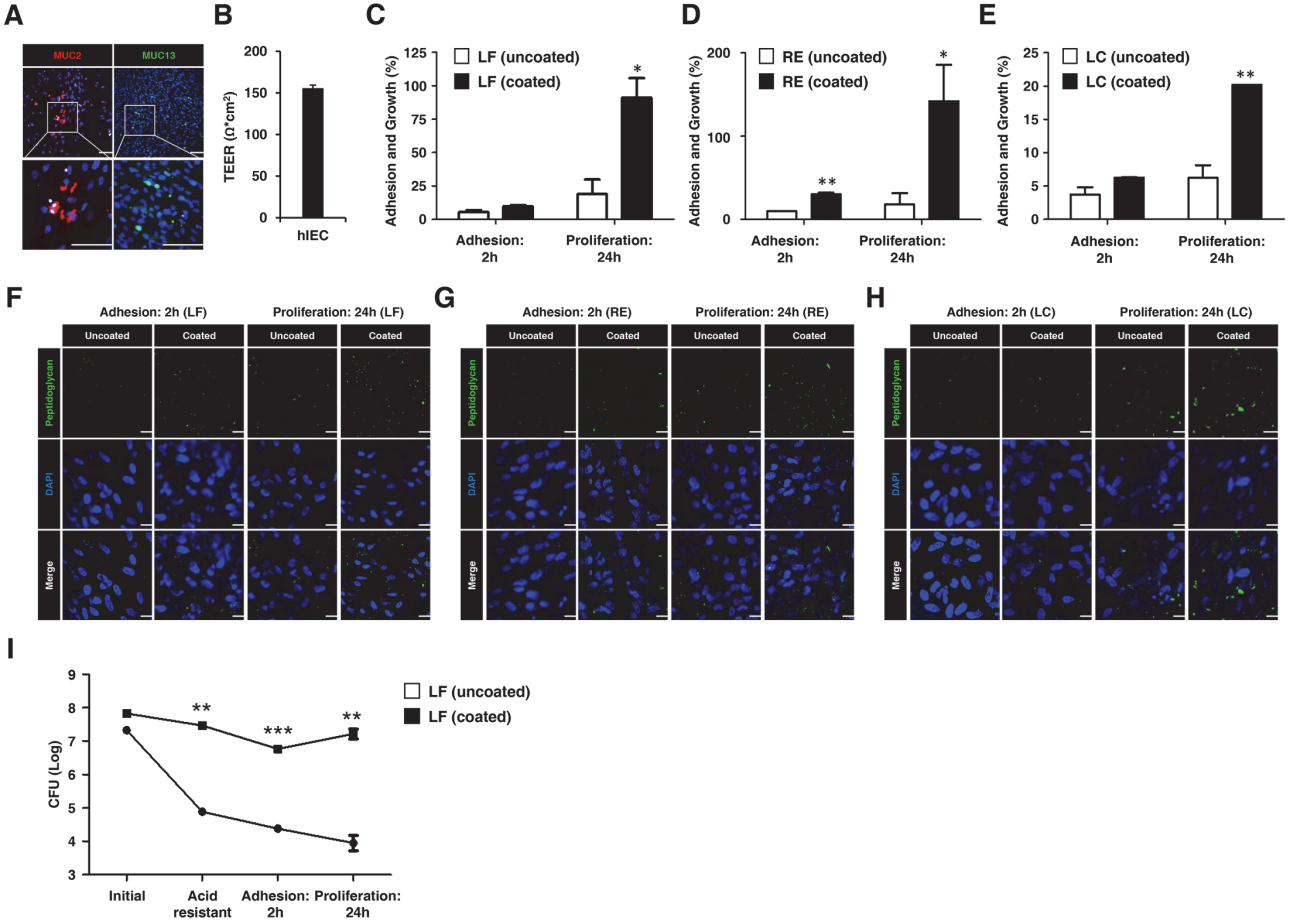
Analysis of multilayer coating effect on LAB strains adhesion and proliferation in hPSC-derived hIECs. (**A**) Representative images of MUC13 expression in hIECs. Scale bar: 100 μm. (**B**) TEER values of hIECs (*n* = 3). (C-E) Analysis of adhesion ability in relation to the initial number of (**C**) LF, (**D**) RE, and (**E**) LC, at adhesion (2 h) and proliferation (24 h) in hIECs (*n* = 4). (F-H) Representative fluorescence images of (**F**) LF, (**G**) RE, and (**H**) LC at adhesion (2 h) and proliferation (24 h) in hIECs. Green fluorescence indicates bacterial peptidoglycan. Scale bar: 20 μm. (**I**) Analysis of adhesion (2 h) ability and proliferation (24 h) of LF in hIECs, after passing through gastric juice. The two groups were compared by a two-tailed *t*-test (*n* = 4; *p < 0.05, **p < 0.01 and ***p < 0.001).

To analyze the adhesion and proliferation potential of CS on normal hIECs, a CFU assay and immunofluorescence staining were performed. The gel-forming mucin (MUC)-2 as the major structural component, and MUC13, which is enriched in mature intestinal epithelium [20], were expressed in normal hIECs with a goblet cell population (Fig. 4A). The transepithelial electrical resistance (TEER) value representing monolayer integrity, was 155.33 ± 2.33 Ohm × cm^2^ (Fig. 4B). The initial adhesion ability of the CS group was found to be higher than that of UCS (Fig. 4C-4E). The adhesion of coated-RE, LF, and LC was 30.2%, 9.7%, and 6.2%, respectively. In contrast, the corresponding adhesion ability of the uncoated-RE, LF, and LC was 9.8%, 5.4%, and 3.7%. Furthermore, the proliferation capacity of the CS group was also significantly higher than that of UCS (Fig. 4C-4E). Specifically, the proliferation of coated-RE, LF, and LC was 127.2%, 91.0%, and 20.3%, respectively, while in the UCS group, these values were 21.8% (RE), 18.9% (LF), and 6.2% (LC), in turn. In the fluorescent images, the CS group displayed a higher number of bacteria adhering to the surface of hIECs compared to UCS (Fig. 4F-4H). To investigate the protective effect of the RGDF-based multilayer coating in simulated gastric juice, CS and UCS samples were passed through an acidic solution and co-cultured with hIECs (Fig. 4I). Based on our observations, the survival ability of bacteria in the CS group was significantly enhanced compared to UCS (Fig. 4I). Furthermore, the coated probiotics attached to hIECs at a significantly higher rate relative to the UCS group, while their proliferation capacity also being improved (Fig. 4I). The uncoated- and coated-LF adhered to hIECs at a measured bacterial count of 2.43 × 10^4^ and 8.93 × 10^7^, respectively. After the adhered strains were incubated for a further 24 h, the measured viable cell numbers for uncoated- and coated-LF were 9.53 × 103 and 1.69 × 10^7^, respectively, indicating a higher proliferation rate of the bacteria in the latter group. Taken together, these results showcased the protective effect of the RGDF-based multilayer coating, as acid resistance, along with the adhesion and proliferation capacities, were significantly enhanced in the CS group compared to UCS, in the normal human intestine-mimicking model system.

## Discussion

The administration of probiotics is associated with positive outcomes for the host. To exert their beneficial effects following oral administration, commensal microbiota induce positive changes by successfully establishing viable long-term populations in the intestine and interacting with the resident gut microflora, or by activating enterocytes or other intestinal microbes via produced metabolites. In this regard, it is important to determine how well LAB can survive in gastric juice and successfully colonize the intestine further downstream. We have previously reported that a multilayer coating comprising of tara gum, basic amino acids (L-histidine, L-arginine, and L-lysine), rice protein powder, and RGDF, increased the survival rate of LAB under extreme acidic conditions as well as their subsequent adhesion capacity [2]. As the intestinal mucus layers consist of negatively charged glycoproteins, crosslinks can be formed through hydrophobic interactions [21]. Kwon *et al*. determined that probiotics have a higher adhesion ability due to their hydrophobic coating [22]. Rice protein peptides that are used as coating materials, contain hydrophobic amino acids at their N-terminus [23]. It has been shown that lysine is able to effectively mitigate the negative impact of dehydration stress on the adhesion ability of probiotic strains, that is caused during the lyophilization process, when a lysine-based mixture is treated with freeze-dried probiotics [24]. As such, based on the evidence from literature as well as our own findings, it is likely that the high adhesion rate observed in the CS group was at least in part owed to the hydrophobic characteristics and the protective effect of the lysine present in the coating material used. In this study, the effectiveness of the multilayer coating method was demonstrated only for three species isolated from *Panax ginseng*. It could be expected that a positive effect is displayed on most lactic acid bacteria since it has already been confirmed that the materials of this coating have a protective effect against freeze-drying [24], an improved adhesion effect [2], and an effect to increase acid resistance [25] in various strains.

Rodes *et al*. demonstrated how the stability of LAB communities is dependent on the retention time by the human colonic microenvironment [26]. In addition, the prolonged retention of probiotics in the gut facilitates their ability to exert beneficial effects by increasing the size of colonization and the quantities of metabolites [27, 28]. In our present study, we observed that all of the bacteria in the CS group exhibited higher proliferation rates and increased adhesion capacity compared to the UCS controls in each intestinal epithelia model system, following a 24 h incubation. Of note, when *L. plantarum* IDCC3501 was cultured with RGDF, the component was found to promote the growth of IDCC3501 by acting as a proliferation enhancer [29]. Moreover, arginine facilitates the proliferation of *Streptococcus thermophilus* T1C2 by protecting the strain against intracellular acid stress [25]. This may also at least partially explain the increased growth observed in the adherent CS group relative to UCS after 24 h, as components of the multilayer coating could enhance proliferation of these strains. The LAB activate arginine biosynthesis to maintain intracellular pH homeostasis under acidic stress [30]. The improved acid tolerance of LC by the addition of arginine and a similar effect of RGDF-coating on LAB acid resistance, has already been explored in literature [2, 31]. Reuben *et al*. highlighted the survival of probiotics in an acidic environment and adhesion to epithelial cells in the host gastrointestinal tract, as essential factors that determine the efficient colonization of the gut environment by probiotic strains [27].

The mucin layer plays a crucial role regarding the adhesion properties of probiotic bacteria, as it allows cells to attach to the host epithelium through their interaction with the mucin-binding protein, as well as several glycoproteins [32, 33]. However, previous reports have described that traditional human colorectal adenocarcinoma cell lines, such as SW480, Caco-2 and HT-29, do not exhibit normal intestinal epithelia physiology, as they are characterized by the absence of cellular diversity, low mucin synthesis, and differences in TEER values, compared to their in vivo counterparts [34-36]. To overcome these limitations, we chose hPSC-derived hIECs in order to examine the multilayer coating effect of RGDF. The hIECs are human intestine-like model systems consisting of gut cell types such as endocrine, goblet, and Paneth cells, as well as enterocytes. As previously reported, hIECs express mucin-related genes and tight junction factors, which more closely reflect the in vivo intestinal mucus layer and physical barrier functions than conventional Caco-2 cells [14]. These properties account for the increased bacterial colonization in hIECs compared to Caco-2 cells. Gene expression patterns, such as transporter-encoding genes and metabolic enzymes of hIECs, resemble those of the human intestine. The Caco-2 cells by contrast, exhibit low levels of mucin-, transporter-, and metabolizing enzyme-related gene expression, which consequently result in lower bacterial adhesion and non-canonical responses to drug treatment [14]. Furthermore, three-dimensional human intestinal organoids can transition to two-dimensional hIEC monolayers while retaining cellular diversity and function [14]. Therefore, hIECs have been proposed as a novel model system for studying bacterial adhesion after passage through gastric fluid, as they more accurately mimic the in vivo conditions of the human gut. Using this hIEC model, we observed that RGDF-based multilayer coating enhanced both the adhesion ability of LAB strains to the epithelial surface, as well as bacterial growth rates before and after passing through the simulated acidic environment of the stomach (Fig. 4). Since we used the hIEC model which is the most advanced and most similar to the human intestinal environment among in vitro model, if the people ingest the multilayer coated-LAB, it could show more beneficial effects in the intestine than administration of uncoated LAB.

In conclusion, here we demonstrated the effectiveness of a multilayer coat that comprises of tara gum, amino acids, rice-derived natural compounds, and RGDF, in enhancing the adhesion ability, colonization capacity, and acid tolerance of LAB strains. We investigated the multilayer coating effect using a human digestive model-mimicking system, which consisted of hIECs and a gastric acid solution. As our findings suggest, when commensal bacteria are directly ingested, a multilayer coating method can provide a colonization advantage and prolong the retention time of the bacteria by the intestine, thereby increasing the likelihood of beneficial microflora populations being established in the gut.

## Supplemental Materials

Supplementary data for this paper are available on-line only at http://jmb.or.kr.
