# Supplementary material for: Multilayer Coating with Red Ginseng Dietary Fiber Improves Intestinal Adhesion and Proliferation of Probiotics in Human Intestinal Epithelial Models
Source: J Microbiol Biotechnol. 2023 Jul 14;33(10):1309–16. doi: 10.4014/jmb.2305.05013 (PMC10619547; doi:10.4014/jmb.2305.05013)
Supplement: Supplementary file 1 [file jmb-33-10-1309-supple.pdf]

J. Microbiol. Biotechnol.
https://doi.org/10.4014/jmb.2305.05013

Table 1. List of antibodies used in this study.

Antibodies	Company	Catalog No.	Dilution
anti-MUC2	SantaCruz	SC-7314	1:50
anti-MUC13	Abcam	Ab124654	1:100
anti-peptidoglycan	Thermo	MA5-16509	1:100
anti-peptidoglycan	Bio-Rad	7263-1006	1:100
